# Canine visceral leishmaniasis in the metropolitan area of São Paulo: *Pintomyia fischeri* as potential vector of *Leishmania infantum*

**DOI:** 10.1051/parasite/2017002

**Published:** 2017-01-30

**Authors:** Fredy Galvis-Ovallos, Mariana Dantas da Silva, Giulia Baldaconi da Silva Bispo, Alessandra Gutierrez de Oliveira, José Rodriguez Gonçalves Neto, Rosely dos Santos Malafronte, Eunice Aparecida Bianchi Galati

**Affiliations:** 1 Postgraduate Program in Public Health, School of Public Health, University of São Paulo – USP São Paulo 01246-904 SP Brazil; 2 Department of Pathology, Federal University of Mato Grosso do Sul-UFMS Campo Grande 79070-900 MS Brazil; 3 Center of Control of Zoonosis of Bauru Municipality 17032-340 SP Brazil; 4 Institute of Tropical Medicine, University of São Paulo São Paulo 05403-000 SP Brazil; 5 Department of Epidemiology, School of Public Health, University of São Paulo – USP São Paulo 01246-904 SP Brazil

**Keywords:** Vector capacity, Sandfly, *Pintomyia fischeri*, *Migonemyia migonei*, Visceral leishmaniasis

## Abstract

American visceral leishmaniasis is a zoonosis caused by *Leishmania infantum* and transmitted mainly by *Lutzomyia longipalpis*. However, canine cases have been reported in the absence of this species in the Greater São Paulo region, where *Pintomyia fischeri* and *Migonemyia migonei* are the predominant species. This raises the suspicion that they could be acting as vectors. Therefore, this study sought to investigate specific vector capacity parameters of these species and to compare them with those of *Lu. longipalpis s.l*. Among these parameters the blood feeding rate, the survival, and the susceptibility to the development of *Le. infantum* were evaluated for the three species, and the attractiveness of dogs to *Pi. fischeri* and *Mg. migonei* was evaluated. The estimated interval between blood meals was shorter for *Lu. longipalpis s.l*, followed by *Pi. fischeri* and *Mg. migonei*. The infection rate with *Le. infantum* flagellates in *Lu. longipalpis* was 9.8%, in *Pi. fischeri* 4.8%, and in *Mg. migonei* nil. The respective infective life expectancies (days) of *Lu. longipalpis*, *Mg. migonei*, and *Pi. fischeri* were 2.4, 1.94, and 1.68. Both *Pi. fischeri* and *Mg. migonei* were captured in the kennel with a predominance (95%) of *Pi. fischeri*. Considering the great attractiveness of dogs to *Pi. fischeri*, its susceptibility to infection by *Le. infantum*, infective life expectancies, and predominance in Greater São Paulo, this study presents evidence of *Pi. fischeri* as a potential vector of this parasite in the region.

## Abstract (fr)

La leishmaniose viscérale américaine est une zoonose causée par *Leishmania infantum* et transmise principalement par *Lutzomyia longipalpis*. Cependant, des cas canins ont été rapportés dans la région de São Paulo en l’absence de cette espèce, avec *Pintomyia fischeri* et *Migonemyia migonei* comme espèces prédominantes, ce qui suggère qu’elles pourraient agir comme vecteurs. Par conséquent, cette étude a cherché à étudier certains paramètres de capacité vectorielle de ces espèces et de les comparer avec ceux de *Lu. Longipalpis s.l*. Parmi ces paramètres, le taux d’alimentation sanguin, la survie et la susceptibilité au développement de *Le. infantum* ont été évalués pour les trois espèces, et l’attrait des chiens a été évalué pour *Pi. fischeri* et *Mg. migonei*. L’intervalle estimé entre les repas sanguins était plus court pour *Lu. longipalpis s.l.*, suivi par *Pi. fischeri* et *Mg. migonei*. Le taux d’infection par des formes flagellées de *Le. infantum* était de 9.8 % chez *Lu. longipalpis*, 4.8 % chez *Pi. fischeri*, et néant chez *Mg. migonei*. Les espérances de vie infectieuses respectives de *Lu. longipalpis*, *Mg. migonei* et *Pi. fischeri* étaient 2.4, 1.94 et 1.68 jours. Les deux espèces *Pi. fischeri* et *Mg. migonei* ont été capturées dans les chenils avec une prédominance (95 %) pour *Pi. fischeri*. Considérant la grande attractivité des chiens pour *Pi. fischeri*, sa susceptibilité à l’infection par *Le. infantum*, son espérance de vie infectieuse et sa prédominance dans le Grand São Paulo, cette étude suggère que *Pi. fischeri* est vecteur potentiel de ce parasite dans cette région.

## Introduction

The leishmaniases continue to constitute serious health problems worldwide mainly because of the dense human populations inhabiting areas of risk and the lack of available vaccines for these neglected diseases [51]. Further, the dynamics of their transmission is complex [2], involving human populations, several different reservoirs and vectors, and a wide diversity of *Leishmania* species. In the Americas, despite the 521 sandfly species recognized [19], 60 of which have been implicated as *Leishmania* vectors, only a few of them are considered proven vectors of the parasites concerned. Recently, authors of entomological studies based on epidemiological criteria and detection of natural infection by molecular methods have suggested that some sandfly species may also be acting as permissive vectors in the transmission of the various species of *Leishmania* [25]. However, the incrimination of species as vectors involves the analysis of vectorial capacity parameters [39] such as vector density, anthropophily, geographical distribution coincident with that of the pathogen, survival, and vectorial competence [27], and prove that the species is essential for maintenance of transmission in nature and that the reduction in its biting rate also reduces incidence of the disease [38]. Therefore, the assessment of these parameters provides information for the evaluation of the role of species suspected of transmitting a particular pathogen, thus contributing to the identification of potential vectors. In the Americas, *Lutzomyia longipalpis* is considered the main vector of *Leishmania infantum*. However, the occurrence of *Le. infantum* infection in canine and/or human populations in the absence of this sandfly species, as well as the finding of other sandflies naturally infected with this agent have suggested that they are vectors exactly as it occurred with *Migonemyia migonei* in Pernambuco state (Brazil) and northern Argentina [13, 37, 40]. In Cotia and Embu das Artes, municipalities of the São Paulo metropolitan area, canine visceral leishmaniasis (CVL) cases have been reported since 2003, but *Lu. longipalpis* has not been found in the entomological surveys undertaken in these foci [11, 31]. On the other hand, *Pintomyia fischeri* was the most frequent species (~95%) collected in previous studies in Greater São Paulo [3, 36], with *Mg. migonei*, *Psychodopygus lloydi*, and *Evandromyia edwardsi* also being reported [45]. Therefore, the present study seeks to estimate certain parameters of the vectorial capacity of *Pi. fischeri* and *Mg. migonei*, and compare them with those of *Lu. longipalpis*, to analyze the role of these species in *Le. infantum* transmission.

## Materials and methods

### Estimation of the attractiveness of dogs to sandflies

**Figure 1.**
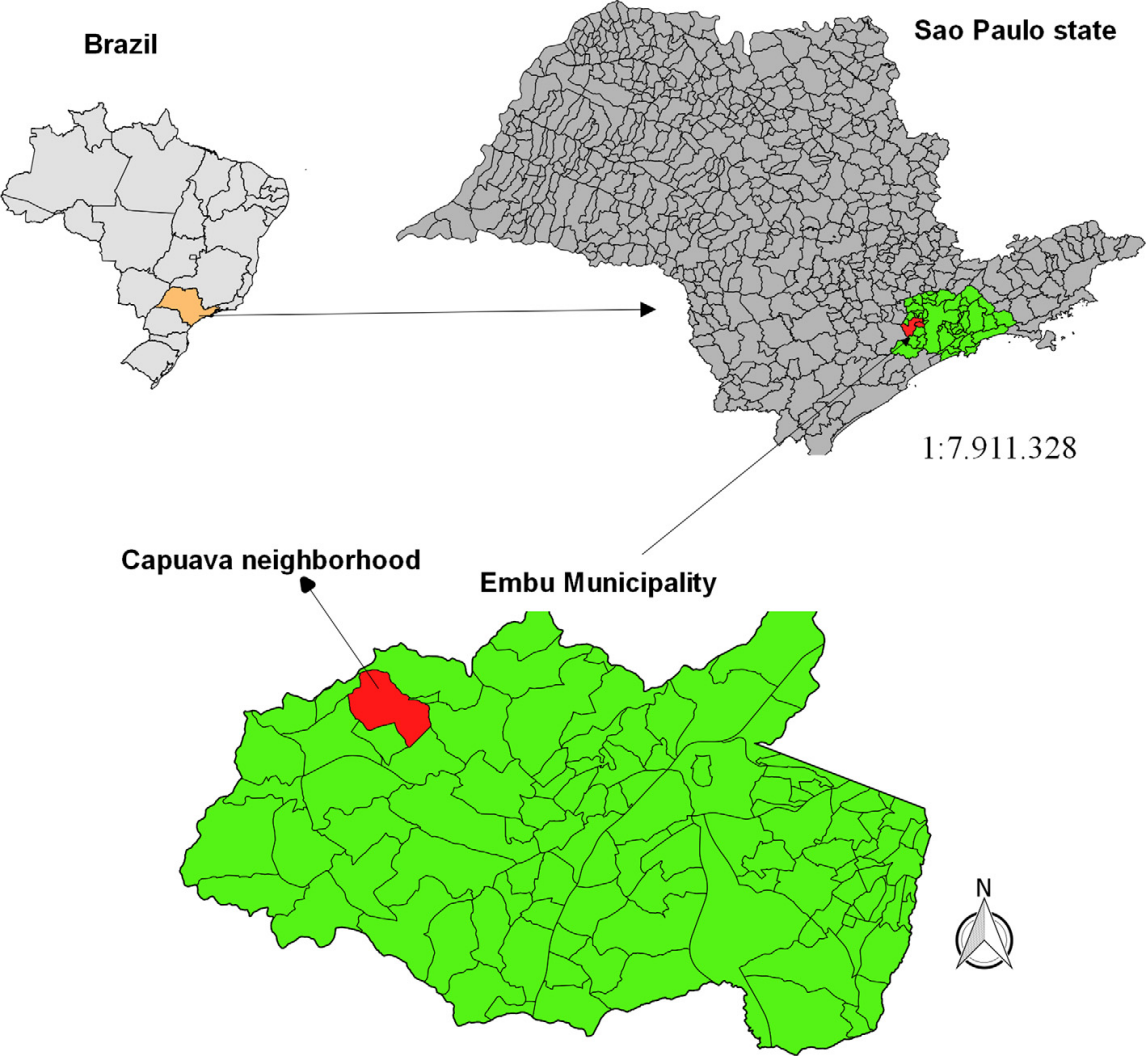
Geographical localization of the study area, Capuava neighborhood, Embu das Artes municipality, São Paulo state, Brazil.

To estimate the density of *Pi. fischeri* and *Mg. migonei* attracted to the domestic dog, 18 collections were performed between October 2010 and December 2011 in a kennel situated at 23° 37.656′ S, 46° 53.164′ W in the peri-urban area (Capuava neighborhood) in the municipality of Embu das Artes, SP (Fig. 1). This municipality was selected in view of the active transmission of *Le. infantum* among the canine population there [42], the absence of the proven vector *Lu. longipalpis* in the region, and the dominance of *Pi. fischeri* and *Mg. migonei* in previous captures undertaken in the same area [6, 45]. The collections were carried out from 6 pm to 11 pm, established in accordance with information obtained in a previous study in the locality indicating that 86% of the sandfly females were captured in this interval.

The sandfly collections were performed with a manual electrical aspirator on the internal walls of the kennel and when the insects landed on the four domestic dogs. To minimize interference from the attractiveness of the human collectors, the aspirations were performed for five minutes in the kennel at 10 min intervals. The captured insects were then killed with chloroform and stored in vials containing 70% alcohol. They were clarified and identified according to the literature [17, 19].

### Evaluation of blood feeding habits on dogs, susceptibility to infection with *Le. Infantum*, and survival

To evaluate these parameters for *Pi. fischeri* and *Mg. migonei*, experiments were undertaken in laboratory conditions and included specimens of *Lu. longipalpis* as a control.

#### Obtaining sandflies

Wild specimens of *Pi. fischeri*, *Mg. migonei*, and *Lu. longipalpis* were captured to obtain F1 specimens*.* However, due to the low yield in colonies of *Pi. fischeri*, wild specimens of this species were collected in the Cantareira Park in São Paulo municipality (a non-endemic area for visceral leishmaniasis (VL)) and used together with those F1 in the first and second experiments. In the last two experiments, only wild specimens were used. The culture of immature forms was undertaken in accordance with the literature [26], at a temperature of 25 °C (±1 °C) and 80% relative humidity.

#### Exposure of dogs

Five experiments in which the insects fed on infected dogs were undertaken. The dogs infected with *Le. infantum* were previously identified according to the clinical and parasitological criteria of the Leishmaniasis Control Program of São Paulo state. Additionally, microscopic and molecular tests were undertaken to confirm the infection. All the dogs used in the experiments manifested symptoms of VL. In each experiment, the dogs were anesthetized with ketamine (15 mg/kg) and xylazine (1 mg/kg) in accordance with the weight of the animal, and then placed in a nylon cage (1 m × 2 m × 1.80 m), already containing the insects (males and females), where they stayed for an hour at a temperature of about 28 °C.

#### Blood feeding on dogs and host-biting habits

The feeding experiments were developed in five stages as follows: the 1st, 2nd, and 3rd with *Pi. fischeri*, *Mg. migonei*, and *Lu. longipalpis* simultaneously, the 4th with *Pi. fischeri* and *Lu. Longipalpis*, and the 5th with *Lu. longipalpis* alone. The first four experiments were undertaken in the Zoonoses Control Center (Centro de Controle de Zoonoses-CCZ) of Bauru municipality and the 5th in the CCZ of Jundiaí municipality, both in São Paulo state. The insects were transported in nylon cages within sealed thermal polystyrene boxes to the experimental laboratories. After 60 min exposure, the dog was removed and the insects captured with a Castro aspirator and conditioned in nylon cages which were placed in the sealed thermal polystyrene boxes and transported to the laboratory. The insects that died during the experiment were collected with tweezers and conditioned in Eppendorf vials for later taxonomic identification. The females were observed until oviposition to estimate the duration of the gonotrophic cycle. After their death, they were clarified in accordance with the literature [17, 19].

After their exposure, the dogs were removed from the cage and underwent euthanasia at the hands of a technician of CCZ. To estimate the frequency of feeding of each species, dog-biting habits were calculated from the expression *a* = AI/GC [20, 21], where:

AI = proportion of females fed on the dog = number of females fed on the dog divided by the total number released into the cage in which the animal was exposed.
GC = Duration of the gonotrophic cycle estimated by the median of the period (in days) between the blood meal on the dog and oviposition.

The relative frequency of the contact of the vector population with the host population was estimated by dividing 1.0 by the value of *a* [20, 21].

A chi-squared test was undertaken to investigate the differences in the proportion of females of each species feeding on the dogs in the experiments and those between the species: *Lu. longipalpis* × *Pi. fischeri*; *Lu. longipalpis* × *Mg. migonei*, and *Pi. fischeri* × *Mg. migonei*.

### Life expectancy after the blood meal

To estimate life expectancy after the blood meal on dogs, cohorts of engorged females of each species were followed up. The daily survival was estimated from the horizontal life table according to the literature [8]. A Cox regression analysis was undertaken to compare the survival of the three populations.

### Susceptibility to infection with *Le. infantum*

In the first experiment, sandfly infection was evaluated by the polymerase chain reaction (PCR) test with a set of rDNA-based primers S4/S12, according to the literature [50]. All the samples of engorged females were followed up and the percentage of positive females was considered after digestion of the blood meal. In the other four experiments, the infection was evaluated by dissection of the females by stereomicroscopy. The rate of infection of each sandfly species was calculated from the number of females with flagellates in their anterior and midgut observed under the microscope, divided by the total of females engorged on the dogs. The identification of metacyclic promastigotes (last-stage infection forms) was undertaken considering the morphology of the flagellates and their development time, according to the literature [4, 25, 30].

### Ethical aspects and biosecurity

This study was approved by the Ethics Committee on the Use of Animals in Research of the Faculty of Medicine of São Paulo University, Process CEP-IMT 057/2009 and by the Biosecurity Committee of the Public Health School of São Paulo University, Protocol 2026.

## Results

### Attractiveness of dogs to sandflies

In the collections undertaken in a kennel in Embu das Artes (SP), 301 specimens (183 females and 118 males) were captured (Table 1). *Pintomyia fischeri* predominated with 95% of the specimens captured and presented the highest dog-attractiveness rates (females/dog/night) in January (8.0), March (5.4), and April (4.5). Only one female of *Mg. migonei* was collected.

**Table 1.** Sandflies captured in kennel by month, sex, and species in Embu das Artes, São Paulo state, Brazil.

Species	*Ev. edwardsi*		*Mg. migonei*		*Pi. fischeri*		*Pi. monticola*		Total		
Month	♂	♀	♂	♀	♂	♀	♂	♀	♂	♀	♂♀
Oct 2010	–	1	1	1	9	18	–	–	10	20	30
Jan 2011	–				12	32	–	–	12	32	44
Mar 2011	–	–	–		–	43	–	–	–	43	43
Apr 2011	–	–	–	–	6	18	–	–	6	18	24
Jun 2011	–	–	–	–	–	21	–	–	–	21	12
Jul 2011	–	–	–	–	–	–	–	–	–	–	0
Aug 2011	–	–	1	4	21	21	–	–	22	25	47
Sep 2011	2	1	2	3	63	12	–	–	67	16	83
Oct 2011	–	2	–	–	–	–	–	2	–	4	2
Dec 2011	–	–	–	–	1	4	–	–	1	4	5
Total	2	4	4	8	112	169		2	118	183	301

### Blood feeding habits on dogs

A total of 993 females and 715 males were used in the experiments, in all of which *Lu. longipalpis* presented the greatest proportion of females which had fed on the dogs. For *Lu. longipalpis* the proportion of dog-blood meals varied from 67% to 83%. For *Pi. fischeri* the blood-meal proportion varied from 46% to 72% and for *Mg. migonei* from 52% to 70%. We observed that under the same exposure conditions, *Lu. longipalpis* presented the highest and most significant proportion of dog-blood meals, while *Pi. fischeri* and *Mg. migonei* presented similar proportions. Only for *Pi. fischeri* were significant differences observed between the proportions of females fed during the experiments (Table 2).

**Table 2.** Number of females of *Lu. longipalpis*, *Pi. fischeri*, and *Mg. migonei* fed on exposed dogs and feeding rate estimated under experimental conditions and comparison of the results of the χ^2^ test between the experiments and between the species.

Experiment	*Lu. longipalpis*			*Pi. fischeri*			*Mg. migonei*		
	Exp	Fed	Feeding rate	Exp	Fed	Feeding rate	Exp	Fed	Feeding rate
1	63	45	0.71	78	49	0.64	70	49	0.70
2*	65	46	0.71	72	33	0.46	23	12	0.52
3	48	32	0.67	145	78	0.53	82	47	0.57
4	110	91	0.83	201	144	0.72	–	–	–
5**	36	30	0.83	–	–	–	–	–	–
Total	322	244	0.76	496	304	0.61	175	108	0.62
χ^2^	7.14; *df* = 4; *p* < 0.05			19.85; *df* = 3; *p* > 0.05			3.6; *df* = 2; *p* < 0.05		

*Lu. longipalpis* × *Pi. fischeri*; χ^2^ = 18.54; *df* = 1; *p* > 0.05, *Lu. longipalpis* × *Mg. Migonei*; χ^2^ = 10.81; *df* = 1; *p* > 0.05, *Pi. fischeri* × *Mg. migonei*; χ^2^ = 0.008; *df* = 1; *p* < 0.001.

* Induced blood feeding.

** Allochthonous canine case.

The median duration of the gonotrophic cycle of *Lu. longipalpis* was 5 days, of *Pi. fischeri*, 6 days and of *Mg. migonei*, 7 days. As regards dog-biting habits (a), it was observed that the highest value was recorded for *Lu. longipalpis* 0.15 (0.76/5), followed by *Pi. fischeri* 0.10 (0.62/6) and *Mg. migonei* 0.09 (0.61/7). Thus, with a frequency of dog biting of 0.15 it is to be expected that one female of *Lu. longipalpis* should take one dog-blood meal every 6.5 days (1/0.15). The corresponding value for *Pi. fischeri* will be 9.7 days and for *Mg. migonei*, 11.4 days.

### Survival of *Lu. longipalpis*, *Mg. migonei*, and *Pi. fischeri* females after a dog-blood meal

**Figure 2.**
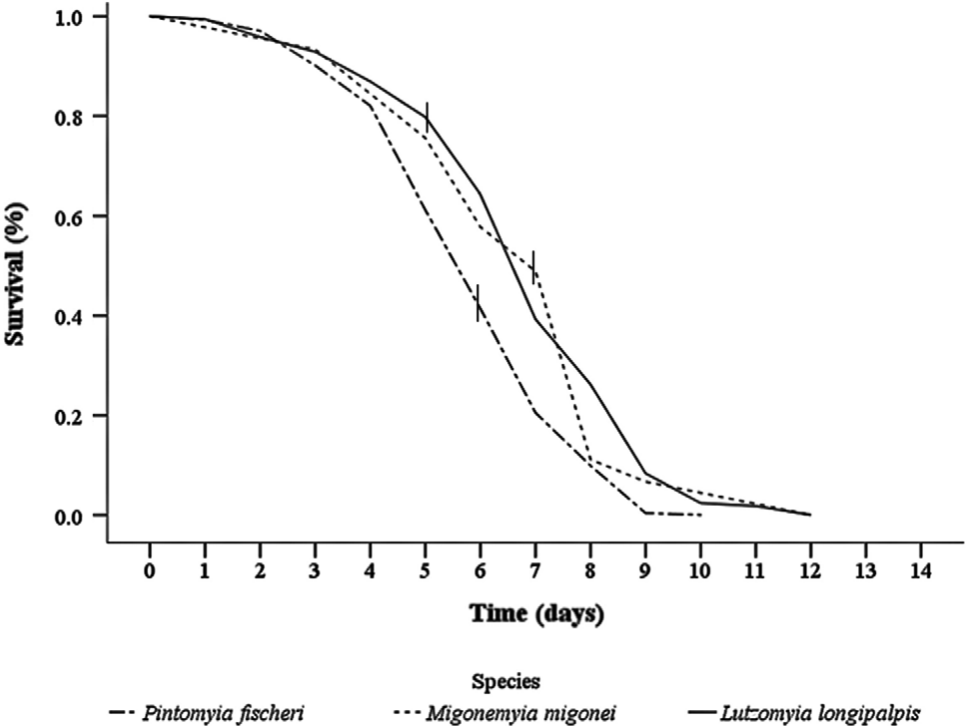
Survival curves of *Migonemyia migonei*, *Pintomyia fischeri*, and *Lutzomyia longipalpis* under laboratory conditions; the vertical line in the curve of each species indicates the median day of oviposition.

Four *Lu. longipalpis* cohorts, with a total of 170 females, were followed up. The median survival of the females of this species after the blood meal was of 7.5 days. At the end of this period of observation of the cohort, it was discovered that at least 1.0% of the females had succeeded in surviving until the 14th day after the blood meal (Fig. 2). Two cohorts of *Mg. migonei*, with a total of 45 females, were followed up. The life expectancy of the females at the beginning of the period of observation was 5.5 days. From the 7th to the 9th day, a fall in the survival curve was observed with an accumulated mortality of 89% by the end of that interval. This high mortality rate coincided with the pre and postegg-laying period (Fig. 2), indicating the possible influence of egg-laying on the mortality of the females. The median survival observed after the infective meal was 7.8 days.

Three cohorts of *Pi. fischeri*, with a total of 263 females, were followed up. The median survival of the cohorts after the infective meal was 5.0 days. Unlike what was observed among the females of *Mg. migonei* and *Lu. longipalpis*, the engorged females of *Pi. fischeri* presented a shorter survival time (Table 3, Fig. 2). However, at the end of the 7th day, a 21% survival rate was still observed, showing that some of the females survived for at least one day after egg-laying.

**Table 3.** Life expectancy and susceptibility to infection with *Leishmania infantum* of *Lutzomyia longipalpis*, *Pintomyia fischeri*, and *Migonemyia migonei* under laboratory conditions.

Species	Parameter						
	Survival of the population		Infection susceptibility to *Leishmania infantum*				
			Molecular analysis		Microscopic observation		
	Life expectancy after blood meal on dog	Life expectancy after completing gonotrophic cycle	Females examined (*n*)	Infection rate (%) after blood digestion	Engorged females	Infected females	Infection rate (%)
*Lu. longipalpis* (*n* = 170)	6.6	2.4	36	31 (85.7)	164	16	9.8
*Mg. migonei* (*n* = 45)	5.5	1.9	32	14 (43.7)	42	0	0
*Pi. fischeri* (*n* = 263)	5.5	1.7	45	11 (24.4)	220	9	4.1

### Susceptibility to infections with *Leishmania infantum*

In the PCR test, the presence of *Leishmania* sp DNA after the blood meal was digested was observed in 85.7% of the *Lu. longipalpis* samples analyzed, and in 43.7% and 24.4% of the samples of *Mg. migonei* and *Pi. fischeri*, respectively.

Flagellate forms of *Le. infantum* were observed in 9.8% of the *Lu. longipalpis* females, 4.1% in those of *Pi. fischeri*, and none in *Mg. migonei.* Metacyclic promastigotes were observed in *Lu. longipalpis* as from the 5th day after the blood meal and massive infection occurred after the 7th day. The development of flagellate forms was observed in *Pi. fischeri* as well as their permanence after the blood-meal digestion and migration to the thoracic gut.

## Discussion

Since the first report in urban areas of São Paulo state of the main vector of *Le. infantum*, *Lu. longipalpis* [12], the first canine case [48] and the first human case [7], the area affected by VL in northwestern São Paulo state has expanded, in association with the dispersal of *Lu. longipalpis*. However, in the municipalities of Carapicuiba, Cotia, and Embu das Artes in the Greater São Paulo region, there are other transmission patterns associated with other sandflies that may be acting in *Le. infantum*, as also observed in other VL foci in Brazil [13, 41]. In the focus of CVL in the Greater São Paulo region, the suspected sandfly species were *Pi. fischeri* and *Mg. migonei*, in view of their dominance [46], but there is no evidence of their vectorial capacity with evaluation of ecological criteria of the vector-parasite and vector-host relationships [21, 27, 38, 39].

### Attractiveness of dogs to sandflies

In the present study, it was observed that the attractiveness of dogs to *Pi. fischeri* was greater than that to *Mg. migonei*; however, it seems that this behavior may be related to the frequencies of the vector species in the area. This observation is reinforced by the data obtained in a previous study in the area, using light traps during 8 captures in 4 different kennels, in which 49 females of *Pi. fischeri* and 4 of *Mg. migonei* were collected (unpublished data), corroborating the frequencies of these species in the study area. Additionally, the greatest dog-attractiveness rate to *Pi. fischeri* was observed during the summer (January and March), which is in accord, respectively, with previous studies [3, 36], indicating the periods of highest frequencies of this species in the Greater São Paulo region. Thus, we conclude that *Pi. fischeri* and *Mg. migonei* are naturally attracted to dogs and therefore the intensity of dogs-sandfly species contact is related to their frequencies in the focus.

There is no doubt that a better quantitative observation of this parameter should be made using several dogs in different localities, in view of the differences in the attractiveness of each animal, environmental variations, and the seasonal distribution of the species. However, some difficulties need to be overcome in determining this parameter, because beyond biological, climatic, and environmental factors, others may also affect the sampling process [47]. Other technical challenges include the need for collections over a long period (night-long and throughout the year) which may lead to the refusal of residents to cooperate and the intervention of the animals’ owners who, with a view to protecting them by applying insecticide in the kennels, thus interfere with the observations. This is especially true in the case of visceral leishmaniasis, since dogs are the reservoirs of the causative agent.

### The habit of blood feeding on dogs

The species’ habit of biting the host is a quantitative measure that enables us to estimate the interval in days between two consecutive blood meals on the host [20]. As it is difficult to obtain these data directly in nature, it may be estimated by means of the proportion of females fed on the host (AI) and the duration of the gonotrophic cycle (GC) [21]. This latter parameter represents an alternative measurement of the biting frequency, when there is gonotrophic concordance. Generally, this parameter is estimated by the examination of the blood ingested by females captured in the intradomicile [21] using molecular or immunohistochemical methods. However, obtaining representative samples of engorged sandflies is no easy task, especially due to the fact that the blood meals on dogs generally take place in peridomiciles, after which the females fly to natural or artificial shelters where the postprandial period occurs. Also, because the samples obtained by this method may be influenced by host density as well as by the spatial and temporal distribution of the species [48], it is difficult to obtain samples of species with low frequencies in the focus.

In this study, we used an experimental method to compare the proportion of females of the three sandfly species feeding simultaneously on dogs, a high proportion of all the females of the three species fed was observed. In the three experiments, *Lu. longipalpis* presented a higher proportion of feeding than *Mg. migonei* or *Pi. fischeri* and the difference was statistically significant. This high degree of cynophile behavior presented by *Lu. longipalpis* corroborates the observations of Camargo-Neves [6] who found 84% of the females of this species collected in peridomiciles in urban areas of Araçatuba municipality (SP) engorged with dog blood. Similarly to our results, a high degree of cynophile behavior was reported for *Pi. fischeri* and *Mg. migonei* in other ecological studies [3, 16]. The high proportion of females here seen feeding on dogs could be influenced by the way in which the animals were exposed (anesthetized), which might favor the females’ biting, since in natural conditions the animals would react (by moving or scratching). However, some studies have indicated that dogs infected with *Le. infantum* may present the asthenia and apathy associated with a loss of movement due to the muscle and motor disabilities which occur during the initial manifestations of the infection [1]. Furthermore, as the dogs remain and sleep in the peridomicile, they are more constantly bitten by sandflies.

As regards the estimate of the duration of the gonotrophic cycle under natural conditions, this information may be obtained by the mark-release-recapture (MRR) technique, but this is difficult because of the normally low recapture rates. With this methodology, a gonotrophic cycle of 3.5 days was estimated for *Nyssomyia neivai* [10]. Under laboratory conditions, a gonotrophic cycle of 7.7 days was estimated for *Mg. migonei*, 6 for *Pi. Fischeri*, and 5 for *Lu. longipalpis*, at a temperature of 25 °C. Thus, with this information regarding the blood feeding rate and the duration of the gonotrophic cycle, the intervals between two blood meals on a dog for *Lu. longipalpis*, *Pi. Fischeri*, or *Mg. migonei* are here estimated at 6.6, 9.7, and 11.5 days, respectively. This time may be shorter in areas with temperatures above 26 °C, since temperature affects the duration of the gonotrophic cycle. Further, after the first gonotrophic cycle, the subsequent ones may be of shorter duration [22]. Our results suggest that under similar conditions of exposure and temperature, the frequency of blood meals of *Lu. longipalpis* on dogs is greater than that of the other sandfly species analyzed and that *Pi. fischeri* will have more frequent contact with the dog than *Mg. migonei*.

### Survival

Although the most widely employed method to estimate the survival of vector insects is the MRR [9, 22], this method presupposes a constant mortality rate independently of age, a fact which has recently been contested in the literature [5, 24, 46]. In this present study, through the life table estimation, a maximum of 10 days post blood feeding was observed for *Pi. fischeri*, 12 days for *Mg. migonei*, and 15 days for *Lu. longipalpis*. In some studies of sandfly dispersion in nature using the mark release recapture (MRR) technique, the recapture time of females recorded varies from 1.5 to 11 days [10, 14, 18, 35]. We observed that the mortality curves of the three species studied presented differences, mainly during and after egg-laying (Figs. 2A–2C). *Pintomyia fischeri* and *Mg. migonei* presented high mortality rates during egg-laying, which has already been described in the literature for other sandfly species [15, 32, 43], and the number of *Pi. fischeri* females which survived (*s* = 0.42) was smaller than that for *Mg. migonei* (*s* = 0.57), this difference being statistically significant (*p* < 0.000). On the other hand, *Lu. longipalpis* presents a greater, statistically significant survival rate (*p* < 0.000) than *Pi. fischeri*. The results of comparison of survival rates for the three sandfly species studied have important implications in terms of their vector capacity and suggest that *Lu. longipalpis* has a longer infective survival time, with the potential of completing at least two gonotrophic cycles. Lainson et al. [28] have succeeded in transmitting *Le. infantum* experimentally to susceptible hamsters by this sandfly species, 7 days after the infective meal. Similar results have recently been described [44]. Therefore, if *Lu. longipalpis* is able to transmit *Leishmania* parasites in that period, our results regarding its survival time suggest that, even in low densities, this species will succeed in maintaining the transmission cycle because of its long infective life (2.4 days). In contrast, *Pi. fischeri* presents a short infective life and for this reason its role as vector could be dependent on the density of the species in the focus. The survival estimation for this species is important in view of the fact that the vector’s survival, as well as its density in relation to the host, influences the efficiency of the agent’s transmission.

### Susceptibility to infection with *Leishmania infantum*

In the experiments to evaluate the susceptibility to infection with *L. (L) infantum*, differences were observed between the results of the parasitological test and those of the PCR. These differences may be related to the delay which occurred between the death of the females and their dissection that could result in the death of the flagellates, which would hinder observation. However, our results showed the susceptibility of *Pi. fischeri* to infection by the automatic vehicle location (AVL) agent as much by the observation of the development of promastigote forms in the gut as by the detection of the DNA in the samples analyzed after the gonotrophic cycle is completed. Although no flagellate forms of the specimens of *Mg. migonei* were observed, the DNA of *Leishmania* was detected in the samples analyzed by PCR in 43.7% of the females that had completed the gonotrophic cycle. Although the positive DNA results do not imply the development and survival of flagellates in the sandfly, recent studies have demonstrated the high susceptibility of *Mg. migonei* to infection with *Le. infantum* [23]. Additionally, this species has been found naturally infected with this parasite species in the state of Pernambuco and in Argentina [13, 37, 40], and our results strengthen the suspicion that this species is a potential vector of the AVL agent.

For the three species analyzed, the rates of experimental infections in the parasitological test were low, which may be related to the differences in the parasite load of the dogs used in the xenodiagnoses and in the design of the experiments. This permitted the spontaneous feeding of the females all over the dog body surface. The infectivity of both symptomatic and asymptomatic dogs for the same species of sandfly is variable [29, 33, 35, 49]. Considering that the species used in the experiments were exposed simultaneously, our results might have been slightly affected by the difference in the infectivity of the dogs and also by the condition of the development of *Leishmania* in each sandfly. New studies must be undertaken to evaluate the parasite load in *Pi. fischeri* and to evaluate the vector competence of this species.

The vector efficiency of a species is related to the compatible duration of the gonotrophic cycle with the extrinsic incubation period (concordance), at least for the first egg-laying. If we assume a similar gonotrophic cycle of 6 days for the three species studied, *Lu. longipalpis* presents an infective survival of 2.4 days, *Mg. migonei* of 1.94 days, and *Pi. fischeri* of 1.68 days. Therefore, in the light of the susceptibility of *Pi. fischeri* and its short infective survival time, this species has the potential to act as a *Leishmania infantum* vector (if its vectorial competence is demonstrated) by means of a density-dependent mechanism.

Our observations are similar to those described for *Pintomyia evansi* in Colombia, suggesting a better adaption of *Le. infantum* in its natural vector *Lu. longipalpis* and a recent adaptation process of this parasite to this sandfly population [34]. The absence of human cases for more than a decade in Embu das Artes could be due to a lower efficiency of *Pi. fischeri* populations in transmitting the VL agent related to a lower infection rate and lower survival. However, these hypotheses should be further evaluated.

In conclusion, in the present study it was demonstrated that in the focus of CVL studied, *Pi. fischeri* presents a higher density than *Mg. migonei*, and also presents higher blood meal rates on dogs. The experimental infection of *Pi. fischeri* with *Le. infantum* demonstrates for the first time its susceptibility to the development of this parasite*.* It is clear that *Pi. fischeri* has a lower efficiency in the transmission of *Le. infantum* than *Lu. longipalpis*, but in the absence of this species, *Pi. fischeri* constitutes a potential vector of the agent of CLV in Greater São Paulo. Nevertheless, its vector competence needs to be further investigated.

## Conflict of interest

The authors whose names are listed declare that they have no conflict of interest in the subject matter or any of the questions discussed in this manuscript.
